# White Paper on European Patient Needs and Suggestions on Chronic Type 2 Inflammation of Airways and Skin by EUFOREA

**DOI:** 10.3389/falgy.2022.889221

**Published:** 2022-06-02

**Authors:** Louise De Prins, Ulrike Raap, Tara Mueller, Peter Schmid-Grendelmeier, Christiane H. Haase, Vibeke Backer, Wytske Fokkens, Linda B. Benoist, Emmanuel Prokopakis, Maria Doulaptsi, Claire Hopkins, Nele Claeys, Thijs Teeling, Lindsay Cypers, Leen Cools, Leif H. Bjermer, Zuzana Diamant, Ulrich Wahn, Glenis Scadding, Claus Bachert, Peter Walther, Sunni R. Patel, Elizabeth Van Staeyen, Peter Hellings

**Affiliations:** ^1^Department of Otorhinolaryngology, EUFOREA Scientific Expert Team Members, Brussels, Belgium; ^2^Department of Otorhinolaryngology, Head and Neck Surgery, Universitair Ziekenhuis Leuven, Leuven, Belgium; ^3^Division of Experimental Allergology and Immunodermatology, University of Oldenburg, Oldenburg, Germany; ^4^Department of Dermatology, Klinikum Oldenburg, University of Oldenburg, Oldenburg, Germany; ^5^Department of Dermatology, Allergy Unit, University Hospital of Zurich, Zurich, Switzerland; ^6^Department of Otorhinolaryngology Head and Neck Surgery and Audiology, Rigshospitalet, Copenhagen University, Copenhagen, Denmark; ^7^Department of Otorhinolaryngology, Head & Neck Surgery, Academic Medical Center (AMC), Amsterdam, Netherlands; ^8^Department of Otorhinolaryngology-Head and Neck Surgery, University of Crete School of Medicine, Heraklion, Crete, Greece; ^9^Department of Otorhinolaryngology, Guy's and St Thomas' Hospital, London Bridge Hospital, London, United Kingdom; ^10^EUFOREA Patient Advisory Board Chairs, Brussels, Belgium; ^11^Upper Airways Research Laboratory, Department of Otorhinolaryngology, University of Ghent, Ghent, Belgium

**Keywords:** atopic dermatitis, asthma, chronic rhinosinusitis, nasal polyps, Type 2 inflammation, quality of life, unmet needs

## Abstract

**Background:**

Type 2 inflammation underlies the chronicity of disease in subgroups of patients with asthma, chronic rhinosinusitis with nasal polyps (CRSwNP) and atopic dermatitis (AD), that often co-exist. Although several studies have investigated the unmet needs of asthma, AD and CRSwNP as such, little is known about the similarities and differences in experiences and perspectives of the current management of patients with comorbid Type 2 inflammatory diseases.

**Aims:**

To improve insight into the common and organ-specific needs of patients with Type 2 inflammation and comorbidities, allowing the formulation of recommendations to better address these needs in the future.

**Methodology:**

This qualitative study was conducted between July 2021 and December 2021 using semi-structured face-to-face or telephone interviews with patients suffering from year-long severe chronic Type 2 inflammation and at least one co-morbid inflammatory condition. Seven participating academic centers in Europe interviewed asthma (Copenhagen and Leuven), CRSwNP (London, Amsterdam and Crete) and/or AD (Oldenburg and Zurich) patients on patient characteristics, disease severity, shortcomings of current care pathways and suggestions for improvement of care. Transcripts were analyzed using an inductive thematic analysis approach.

**Results:**

Eighty-one patients with severe Type 2 inflammation and comorbidities were interviewed. Similar needs were recognized by patients with Type 2 inflammation, with both a lack of coordination in care and a lack of a real cure reported as being most frustrating. However, several needs are specific to asthma, CRSwNP and AD. Suggestions for improvement of care were generic across diseases, such as the implementation of a multidisciplinary approach, the improved facilitation of access to better treatments, the increase of general awareness on disease burden, and better educational programs for healthcare providers and patients. Of note, patients with CRSwNP also stated the need for alternatives to sinus surgery, whereas patients with asthma requested better medical care to prevent exacerbations and patients with AD would warmly welcome the reimbursement of emollients.

**Conclusion:**

Patients with asthma, CRSwNP and AD have shared unmet needs that need to be addressed by physicians, the academic community and health policy makers. This survey provides unique recommendations made by patients for the implementation of better care.

## Introduction

Chronic rhinosinusitis with nasal polyps (CRSwNP), asthma and atopic dermatitis (AD) are chronic inflammatory diseases of the upper airways, lower airways and the skin, respectively. The majority of these patients share a common pathogenesis, called Type 2 inflammation, with different T-helper 2 and ILC2 (type 2 innate lymphoid cells) cytokines driving the chronicity of inflammation in the different organs (1, 2). Globally, the prevalence of Type 2 inflammatory diseases is increasing with CRSwNP, asthma and AD currently affecting up to 3%, 1–18% and 10% of the population respectively (3–5). Patients with CRSwNP, asthma and AD frequently have Type 2 comorbidities, with asthma occurring in up to 65% of patients with CRSwNP and in up to 49.8% of patients with AD (6–8). Although limited studies have investigated the prevalence of comorbid AD and CRSwNP, it is known that there is somewhat an association between the diagnosis of AD and the development of CRSwNP (1, 9). Furthermore, the severity of CRSwNP, asthma and AD is higher when concomitant Type 2 comorbidities exist, with suboptimal outcomes in care if comorbidities are neglected (10, 11), including high recurrence rates and the frequent need for systemic corticosteroids (5, 12). In those patients with severe disease, CRSwNP, asthma and AD have a high symptom burden with significant impairment on quality of life. Aside from the typical sinonasal, bronchial and dermatological symptoms, the presence of Type 2 chronic inflammation is characterized by sleep disturbance, absence from work, social isolation, learning difficulty and increased incidence of depression (3, 5, 13). Having nearly reached epidemic proportions, Type 2 driven diseases account for an important portion of overall healthcare costs (14, 15).

Preliminary qualitative research has reported on the reduced quality of life due to the presence of asthma, CRSwNP and atopic dermatitis as well as on patients' frustrations with perceived inadequate treatment and lack of coordinated care (16–19). Limited information is currently available on the shared unmet needs in care pathways of patients with comorbid Type 2-driven diseases. In parallel, advocacy efforts related to asthma, chronic sinus disease and atopic dermatitis as comorbidities have neither addressed the patient needs, nor their suggestions for better care. EUFOREA is an international non-profit organization aiming to help healthcare providers implement optimal care for patients suffering from chronic respiratory diseases (20, 21). In that context, EUFOREA has recently published a paper to address the unmet needs of European patients with CRSwNP, including the benefits and shortcomings of care focused on the upper airways (17). The Patient Advisory Board of EUFOREA aimed to expand the previous CRSwNP initiative on unmet needs in CRSwNP and include the view of patients with asthma and AD, providing a better overview of shortcomings of care in those patients who often present with comorbidities resulting in this White Paper.

This qualitative study amongst a sample of European patients aimed to elucidate the disease burden and to explore patients' perspectives of current care strategies with focus on their perceived issues of care and suggested ways to improve the disease journey. In that sense, this White Paper intends to contribute to the design of improved care strategies for patients suffering from Type 2 inflammation and comorbidities. Therefore, our study aim is to improve insight into the current needs of patients with Type 2 inflammation and to introduce recommendations to address these needs.

## Materials and Methods

### Study Design and Participants

This study was carried out using an inductive qualitative research design with semi-structured interviews (22). Specialists from different European respiratory (Copenhagen, Leuven), dermatology (Oldenburg, Zurich) and ENT (Ear, Nose, Throat) (Amsterdam, Crete, London) departments were invited by email to request their willingness to participate in a qualitative study to explore the unmet needs related to Type 2 inflammatory conditions. Specialist centers were selected based on the willingness of the local expert leaders to participate. The initial enrollment target was 12–15 interviews per center, with plans to continue interviews until thematic saturation was achieved, meaning no new themes were emerging. A purposive sampling strategy was used to ensure the recruitment of patients with a wide range of demographic, socio-economic and health status characteristics.

Adult patients meeting the following 4 criteria were selected for the face-to-face or telephonic interviews: (1) diagnosis of moderate to severe Type 2 disease (asthma, AD or CRSwNP), (2) disease history of more than 5 years, (3) presence of at least one Type 2 comorbidity [asthma, AD, CRSwNP and/or allergy (skin-, food- or respiratory allergy)], and (4) age between 18 and 75 years. The diagnosis of moderate to severe disease was confirmed by an ENT, respiratory or dermatology specialist using disease-specific tools (moderate to severe asthma defined as ACQ > 1.2, moderate to severe AD defined as SCORAD ≥ 26 and/or IGA > 3, moderate to severe CRSwNP defined as SNOT22 > 45) (3, 5, 23).

### Data Collection and Analysis

Data was collected between July 2021 and December 2021. PH and LDP developed a semi-structured interview guide in English which was assessed and refined by the whole research team (24). This interview questionnaire provided clearly worded, patient-oriented and open-ended questions in order to encourage an in-depth and spontaneous description of patients' perspectives. The questions (presented in Appendix 1) focused on the patients' views and experiences regarding their disease and care pathway. Baseline respondent characteristics, disease duration, current treatment and corticosteroid use were also requested. In addition, participants scored the severity of their diseases through a Visual Analog Scale (VAS) from 0 (no symptoms) to 10 (worst imaginable symptoms).

To account for the limiting factor of language barriers, interviews were conducted in the patient's native language, i.e. Dutch, German, French, English, Greek or Danish. Interviews were carried out by a bilingual (fluent in English and the local language) trained local clinical research assistant, who is familiar with the subject matter (asthma, CRSwNP or AD). According to the interview guide, interviews were either conducted face-to-face at the local hospital or *via* telephone.

Responses were anonymised, recorded and transcribed verbatim or typed out immediately depending on the experience of the interviewer. The minutes were translated into English by the interviewer and subsequently forwarded to an independent researcher (LDP; medical student, familiar with Type 2 inflammation and experience in qualitative research, not previously known to the participants) who was primarily responsible for the qualitative analysis. Inductive thematic analysis was conducted from October 2021 to December 2021 (9, 25, 26). LDP familiarized herself with the data by carefully reading and reviewing all transcripts. An initial list of ideas was noted. Recurrent patterns and meaningful concepts were identified and labeled with codes. Coding decisions were discussed with PH. Once the codebook was finalized, NVIVO R1-software was used to facilitate the coding process and manage data. Data saturation was reached, with no further interviews conducted. Codes were clustered, linked, refined and re-labeled leading to the identification of interesting overarching themes and subthemes. The qualitative analysis and themes were reviewed, and after team discussion, 4 major themes were identified. All interviews were re-read by LDP for a final evaluation of the accuracy and comprehensiveness of the storyline. Finally, a report of the essential research findings was composed including significant quotes to illustrate major ideas in each theme or subtheme.

### Ethical Considerations

The study was carried out following the requirements of the local ethical committee guidelines of every participating center (University of Copenhagen, University of Leuven, University of Oldenburg, University of Zurich, London Bridge Hospital, University of Amsterdam, University of Crete). All participants were briefed about the study objectives and oral informed consent was provided prior to initiating the interview.

## Results

Overall, seven university centers in seven European countries (Belgium, The Netherlands, United Kingdom, Greece, Switzerland, Germany and Denmark) agreed to participate in this survey. A total of 81 patients were interviewed resulting in a sample with diverse patient characteristics (shown in Table 1). The interviews lasted between 10 and 60 min. Transcripts lacking baseline characteristics and comorbidities were excluded. Ultimately, 70 interviews were included for data processing including 28 CRSwNP patients, 21 asthma patients and 21 patients with a principal diagnosis of AD.

**Table 1:** Overview of patient characteristics.

**Patient Characteristics**	**Asthma**	**AD**	**CRSwNP**
**Age**	21	21	28
(mean, range, in years)	48 (25–73)	38 (20–74)	50 (28–71)
**Gender**	
Female	15	8	12
Male	6	13	16
**Comorbidity**	
Asthma	–	11	23
AD	2	–	2
CRSwNP	21	15	–
Allergy	4	12	14
**Diagnosis period**	
(mean, range, in years)	18 (5–39)	25 (3–56)	17 (2–57)
**Severity of disease**	
(mean, range, on scale 0–10)	6 (0–9)	7 (3–10)	6 (0–10)
**Current treatment**	
Periodically OCS	17	12	16
Biologic treatment	0	3	6
**Nationality**	
Belgian	11	–	–
Danish	10	–	–
Dominican	–	–	1
Dutch	–	–	12
German	–	15	–
Greek	–	–	2
Spanish	–	–	1
Syrian	–	–	1
Swiss	–	6	1
UK	–	–	10

The mean age of participants was 46 years, with equal gender distribution (50% male) and with 10 different nationalities represented. The disease history ranged from 2 to 57 years with a mean of 20 years. The mean severity of disease was 6 determined by the Visual Analog Scale (VAS). Fifty-five study patients suffered from (comorbid) asthma, whereas 64 had (comorbid) CRSwNP, 25 had (comorbid) AD and 30 had a comorbid allergy. Patients with CRSwNP reported asthma as the most frequent comorbidity. Allergy as a comorbidity was also frequently mentioned including allergic rhinitis and food allergy. Sixty-four percentage of respondents used OCS (oral corticosteroids) periodically.

**Figure 1:**
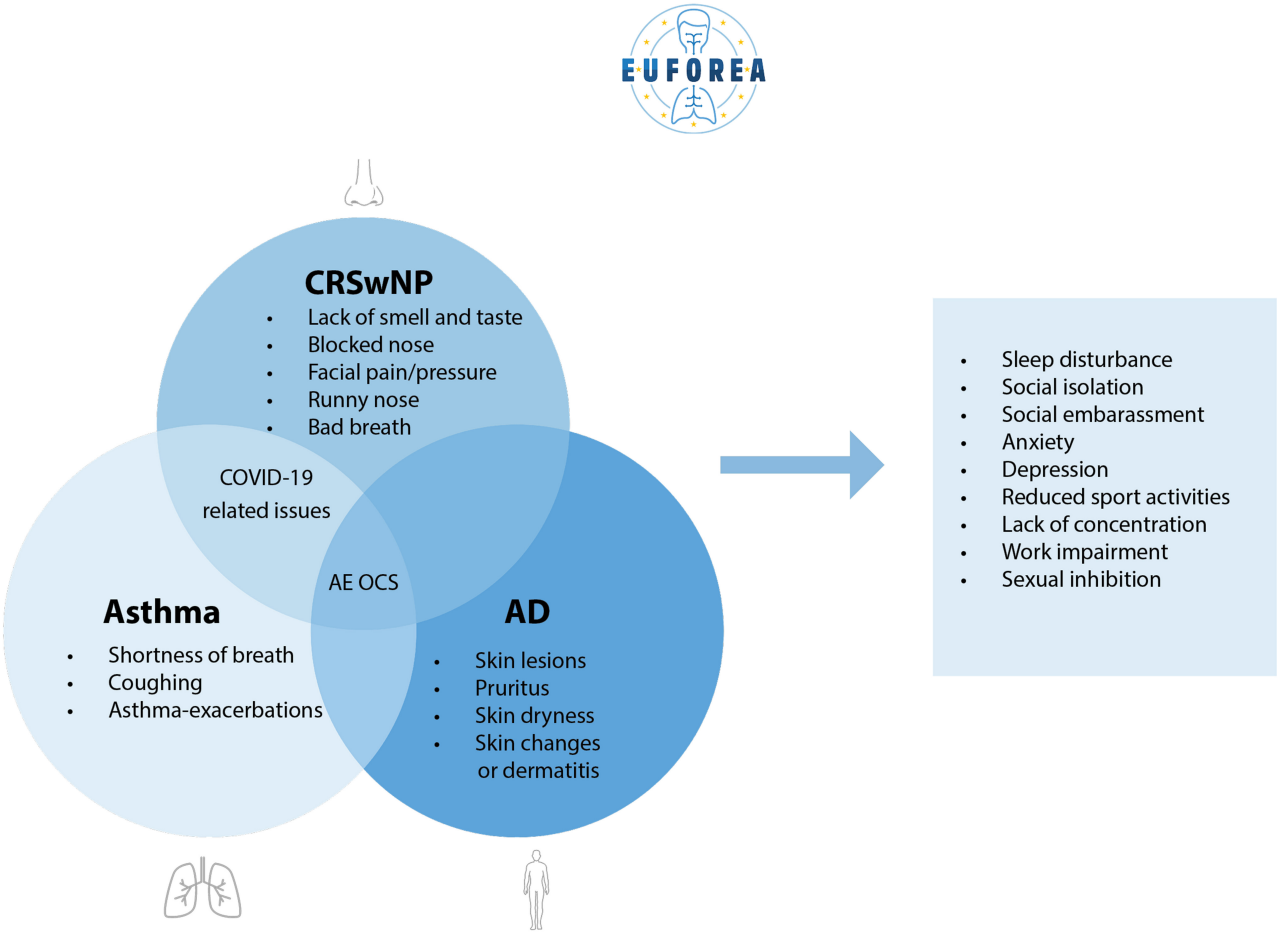
Summary of the burden of Type 2 inflammatory conditions (AD, asthma and CRSwNP), statements ordered by number of times reported.

**Figure 2:**
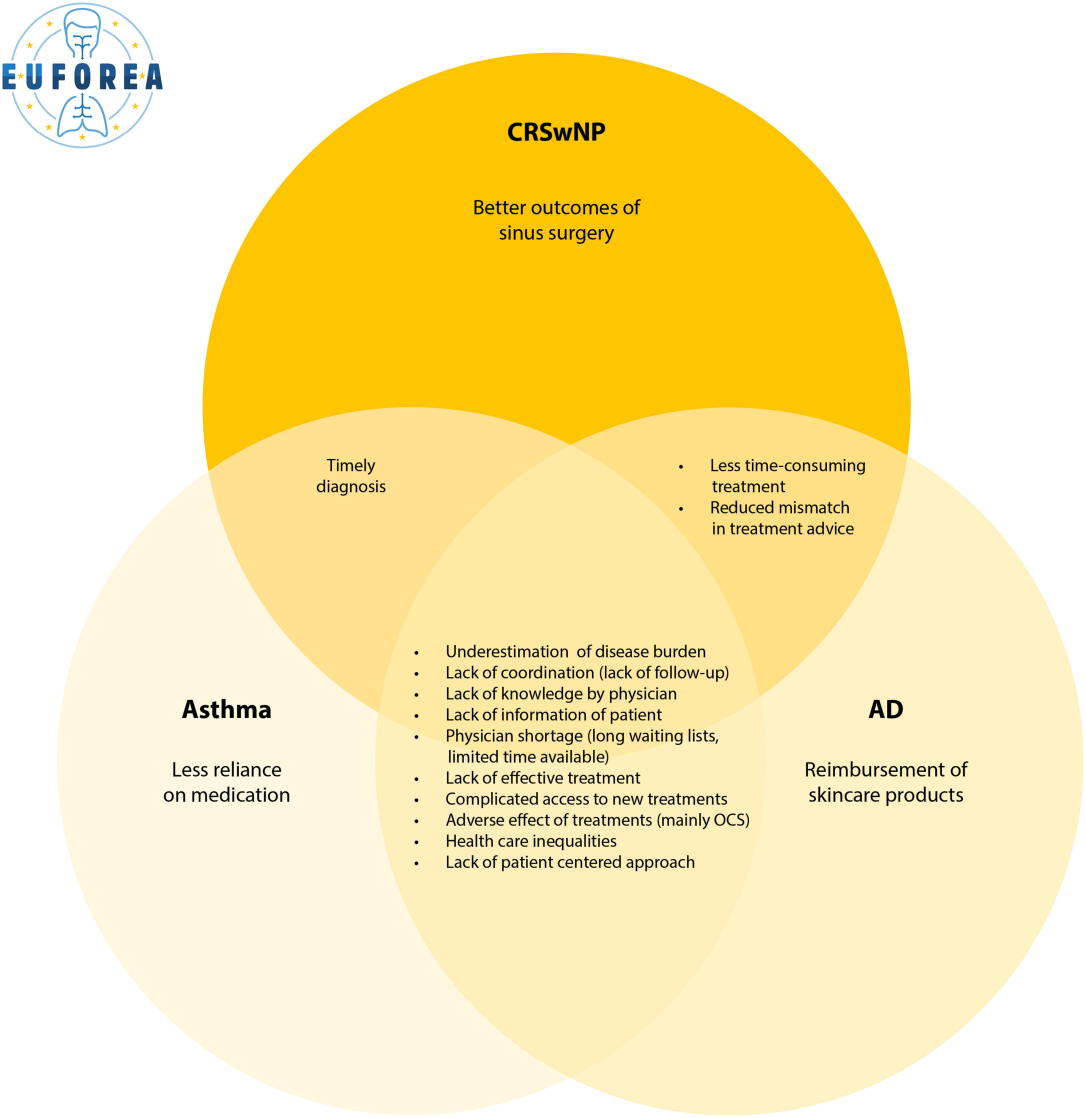
Overview of the perceived unmet needs of current care for patients with Type 2 inflammation, statements ordered by number of times reported.

Interestingly, patients with asthma, AD and CRSwNP expressed mainly similar barriers in and suggestions for their disease journey. Despite disclosing frustrations mentioned by most interviewees, we noticed that most of the interviewed patients reported overall satisfaction with current health care, at a tertiary center level, but with a margin for improvement. Based on the responses, four main themes were identified: (1) quality of life impairment, (2) treatment dissatisfaction, (3) need for coordinated care, and (4) potential solutions. A summary of the burden of Type 2 inflammatory conditions is shown in Figure 1. Figure 2 represents an overview of the perceived unmet needs.

### Quality of Life Impairment

#### (In)Visibility of Disease

Nearly one third regretted the underestimation of the burden of their disease. Type 2 inflammatory diseases, in particular asthma and CRSwNP, are often invisible conditions. There may be no visible signs of the debilitating symptoms that patients face daily. According to respondents, this may partly explain the lack of understanding, recognition, empathy and support by primary care, family and society.

“*Other people can't see on the outside of me how much the illness affects me. It is not like having a broken leg. I would like that people could be able to see how I am feeling.” (asthma patient)*

“*I am constantly confronted with people's lack of understanding. Even within my own family, I am not taken seriously.” (asthma patient)*

On the other hand, almost half of the participants reported a feeling of social embarrassment due to the visibility of their disease. Skin lesions, scratching, bad breath, runny nose, sneezing, coughing, shortness of breath and nasal voice are common symptoms that cause embarrassment in the presence of other people. In addition, some reported that CRSwNP and asthma can mimic COVID-19 symptoms. They were regularly confronted with stares and questions about the contagiousness of their disease.

“*I have swollen eyes and erythema. I prefer to stay away from the public.” (AD patient)*

“*I don't want to look at myself in the mirror.” (AD patient)*

“*It is harder to establish new contacts due to my appearance.” (AD patient)*

“*People do not come and sit next to me on the bus. They think I have COVID-19.” (CRSwNP patient)*

#### Sensory Loses

Participants suffering from CRSwNP perceived an altered experience of the world. Impaired hearing and loss of smell and taste create a disconnection from the environment. The same group explained that missed sensory impressions from their environment had the most direct impact on their mental well-being with anxiety, depression and social isolation as reported consequences. In parallel, a loss of pleasure in eating, cooking and other food-related activities were commonly described.

“*The fact that I have no sense of smell and taste leads to less pleasure in social occasions. This uses a lot of energy.” (CRSwNP patient)*

“*I miss a lot of impressions from the environment due to reduced hearing and absent smell. Eating out is no longer fun.” (CRSwNP patient)*

#### Reduced Activities

The limitations in daily functioning that Type 2 inflammation can cause were reported as a disabling and significant impact. Besides the social embarrassment, participants highlighted the impact of physical discomfort and impairment on sleep, work, sports, hobbies and social activities.

##### Physical Discomfort

Respondents mentioned that they have had to permanently adapt their way of life to avoid triggers that could lead to disease deterioration. In participants with comorbid asthma, shortness of breath and coughing, they have limited themselves in undertaking physical activities and wearing a facial mask. Patients with CRSwNP expressed the heavy impact of sensory loss, excess of mucus, blocked nose and facial pain on their work and social life. For AD, itching was the most evident symptom leading to reduced social contact overall. Additionally, the mention of burning and itching skin affected their choice of clothing as many types of fabrics can make the symptoms unbearable.

“*I have severe facial pain - it's the worst thing I've ever experienced.” (asthma patient)*

“*I have the feeling of having a constant hangover.” (asthma patient)*

Patients with asthma and CRSwNP have difficulties with physical exercise because of shortness of breath and facial pain, whereas patients with AD reported pruritus as the most limiting factor.

“*I have decreased contact with friends due to strong pruritus in the evening and night. I am not able to attend evening events. Long car rides and sport lead to pruritus.” (AD patient)*

“*Shortness of breath prevents me from doing physical activities.” (asthma)*

“*When I do something, I get tired much faster. Then I have to sleep more.” (asthma patient)*

Lastly, sexual inhibition was a recurring reported theme due to physical discomfort. Respondents noted that diverse symptoms such as skin lesions, pruritus, runny nose, headache and malaise made them feel uncomfortable in their bodies and less willing to take part in sexual activities.

“*If I turn my head down and I raise my head up again - then huge amounts of watery fluid runs out of my nose. It also affects my quality of life when I have sex because watery fluid rushes out of my nose. That is not nice.” (asthma patient)*

##### Sleep Impairment

The interviews revealed that patients with Type 2 inflammatory considered fatigue as an important reason for their reduced quality of life. Nervousness as a side effect of OCS, itching, runny nose and blocked nose resulted in sleep disturbance. Participants mentioned that a lack of sleep and a lack of concentration due to clinical symptoms were an important issue when it came to work and social performance. The majority felt that they were less able to fulfill their tasks. Some even reported absence from work. This was certainly the case for patients with CRSwNP who underwent surgery regularly. Social engagements such as dinners were often canceled due to exhaustion. After work, many felt exhausted, had no remaining energy and felt like putting everything aside for sleep.

“*I never sleep well. It's probably been 10 years and I'm never well rested. The biggest frustration is that I am never feeling fresh – max 80% and often below that*. […] *It has had a very big impact on my social life because I have to say no to a lot of things because of my illness. As an example, my daughter-in-law and grandchildren live in Canada and I don't see them very often, but sometimes I have had to say no to traveling to them because the symptoms have too much impact on me.” (asthma patient)*

“*Working is just about possible. But afterwards, I have no energy left to do social things.” (CRSwNP patient)*

#### Psychological Impact

Comments reflecting the ongoing challenge of living with Type 2 inflammation revealed a major impact on psychological well-being. Anxiety and stress were a prominent theme with various types of causes. Patients voiced concerns regarding contracting COVID-19, the continued risk of asthma exacerbations, the worry of side effects from their current treatment and the fear of other people's opinions. They highlighted the burden of asthma, CRSwNP and AD on their mood. Participants suffered from mood swings and were more irritable. A remarkable number even became depressed. Further, feelings of helplessness, loneliness, frustration, uselessness and not being understood were frequently mentioned.

“*I became introverted. Normally, I am not introverted - I am extroverted. It also gives me the sense of being in a “bubble” and then I shut down.” (asthma patient)*

“*In the past, my illness was not recognised as background for my anxiety. Sometimes, I have been crying in front of other people - I had to fight to be taken seriously. It gave me anxiety, that I was being told there was nothing wrong with me.” (asthma patient)*

“*I do not experience joy in life. I feel depressed.” (asthma patient)*

### Treatment Dissatisfaction

#### Lack of Effective Treatment

A majority of participants pointed out that they felt the efficacy of their treatment was inadequate. They still experienced a major burden of symptoms and recurrence regardless of the correct administration of their medication. This lack of disease control prevented them from living a normal life. Some were anxious that a solution to their condition would never be found. Patients with CRSwNP reported the lack of a sustained effect of sinus surgery as the number one disease-specific shortcoming, given the recurrence of nasal polyps after a varying period of time. Another cited shortcoming by all patients was the lack of preventive treatment, the lack of cure. Participants pointed out that their medication suppressed their symptoms but did not address the cause, hence making them dependent on their medication. In particular, patients with asthma reported they rely on always having to think about emergency medication with panic in case of neglect.

“*Topical steroids are the only treatment option for my disease. They have serious side effects. There is no treatment which addresses the cause of my disease.” (AD patient)*

“*Only symptoms are treatable. They are not treating the root cause of disease. Finding out personal triggers is time-consuming.” (AD patient)*

“*I have been searching for a long time, but no treatment has worked so far.” (CRSwNP patient)*

“*There are no possible preventive actions to put a stop to an outbreak of the disease.” (AD patient)*

#### Limited Access and Funding

The lack of proper reimbursement and complex access to certain therapies were experienced as major barriers in patients' care pathways. This topic was primarily reiterated amongst patients with AD as skincare is not reimbursed. A minority of participants stated that they had experience with the use of biologicals, either by participating in a trial setting, by self-paying or by meeting the eligibility criteria due to the severity of their disease. Patients who participated in a recent trial on biologicals reported that disappearance of symptoms had a significant effect on their quality of life. Some of them subsequently had to reaccept the issues around their health condition as they did not have access to biologics beyond the research project. High treatment costs and the limited availability of treatment options were associated with feelings of helplessness, frustration, anger and tiredness.

“*It's a merry-go-round of non-effective, invasive treatment when the answer is already in front of us. Surgery is invasive, but for many a shot of Dupixent would resolve their symptoms. Dupixent solved 20 years of sickness in 48 hours with limited side effects.” (CRSwNP patient)*

“*Products for basic skincare are not financially covered by insurance. The same applies to antihistamines. The costs are above 500 euro each month.” (AD patient)*

“*I have been taking CS for a long time: now I'm on an anti-IgE medicine Xolair. It works really well but to get it reimbursed I first had to suffer very badly. I had to drop below a certain lung capacity. It was only after my admission to the hospital that I qualified for reimbursement.” (asthma patient)*

#### Burden of Treatment

Besides the treatment costs, the burden of treatment fell broadly into two categories: the adverse effects and the time-consuming aspect of the treatment.

##### Adverse Effects

The reported unpleasant side effects of treatment were primarily associated with OCS and nasal polyps surgery. Patients reported anxiety, depression, lack of concentration, insomnia, osteoporosis, stomach problems and weight gain as OCS-related adverse events. On the other hand, surgery resulted in days lost from work, nausea and pain according to patients with CRSwNP.

##### Time-Consuming Therapy

The time-consuming nature of various Type 2 inflammation therapies was another frequently mentioned theme. Patients with comorbid AD viewed the daily application of emollients as an exhausting and never-ending task. Within the CRSwNP group, nasal rinses with saline were reported as time-consuming as well as the recovery from surgery that could require up to several weeks. In addition, the travel time to a specialized center was described as long and tiring for patients with asthma, CRSwNP and AD.

“*Having to travel to London for specialist care is a negative aspect of my treatment.” (CRSwNP patient)*

“*Rinsing the nose twice a day takes a lot of time and motivation.” (CRSwNP patient)*

### The Need for Coordinated Care

Responses provided detailed insights into the experience of a lack of continuity of healthcare. Primarily, patients with asthma and CRSwNP reported a delay in diagnosis. This was, in their opinion, linked to a lack of knowledge and motivation from their GP (general practitioner). Some participants also experienced this lack of expertise when consulting a secondary care physician. The search for appropriate care was described as long and exhausting. When adequate care was finally found, patients felt that results and treatment plans from tertiary care were not efficiently shared with their GP which contributed to a lack of follow-up. Many reported physician shortages. This was demonstrated both by limited time available during consultations as well as long waiting lists. The latter prevented patients from receiving timely care during an exacerbation.

“*It took at least two years to get a diagnosis. I decided to go to a lung specialist on my own initiative as a result of my symptoms. I had to search for help on my own.” (asthma patient)*

“*I saw an enormous number of specialists. It was a rollercoaster before I met the right people. It took a long time before I felt safe and helped.” (asthma patient)*

“*GPs don't know enough about the disease and so people are suffering unnecessarily for years.” (CRSwNP patient)*

Patients felt disappointed with the lack of management of their comorbidities. In particular, the lack of concern for their mental health was stated. Several highlighted that physicians were only focusing on their field of expertise without considering the patient as a whole. According to some, their individual preferences, triggers that aggravated their disease and specific needs were not taken into account.

“*I need them to see my psychiatric diagnosis too because it all comes together. I need them to see me as a whole person. I also think there's a barrier there in that I'm older. I'm 73 years old.” (asthma patient)*

“*For example, I once had cervical disease (onset of cancer), my gynaecologist told me that because of my upper respiratory disease I was at much higher risk for this. I was shocked that my pulmonologist had never told me this before. So shouldn't we get screened more often?” (asthma patient)*

“*It is also the large change with different doctors. My former doctor listened to me when I said it was time for new surgery, my new doctor does not. I have had 15 operations for polyps, so I know when I need a new one. I know when the polyps are bad and when they are not. I feel that the doctors know how I am feeling.” (asthma patient)*

“*That I am feeling like that I'm “falling between two chairs”- because often if I have an appointment based on one part/symptom to my illness, then it is often the other symptoms which is the worst.” (asthma patient)*

Individuals with AD and CRSwNP often experienced disagreements between physicians leading to confusion and mistrust. Inconsistency within European countries was experienced when it came to management guidelines, reimbursement criteria and availability of medicines.

“*Flixonase nasules are not available in own country and are not reimbursed.” (asthma patient)*

“*I'm very frustrated with the fact that I am constantly sent to other doctors and constantly receive another advice.” (AD patient)*

“*Doctors of different specialities are having disagreements regarding my topical therapy.” (AD patient)*

### Potential Solutions

**Figure 3:**
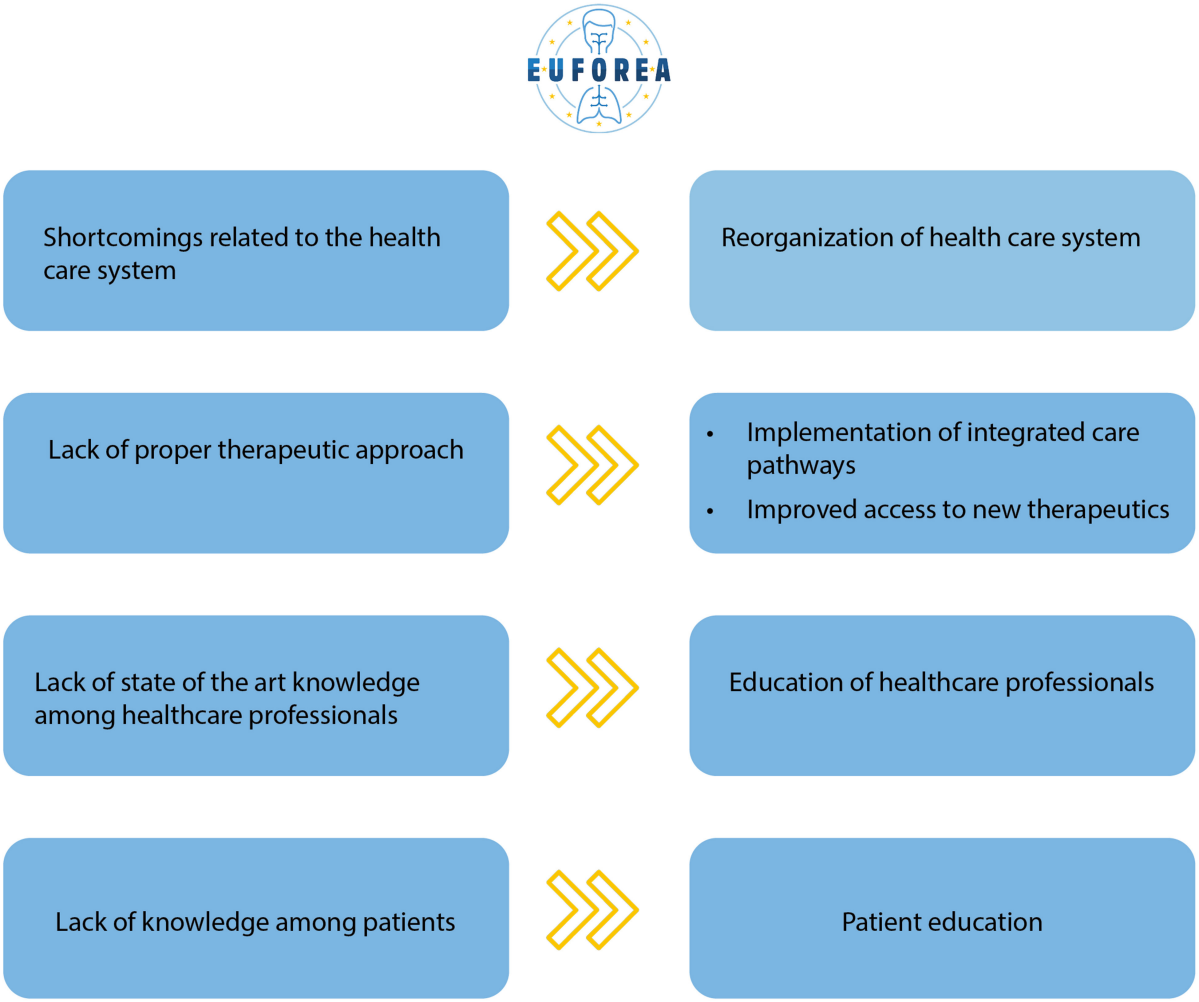
Overview of the 4 key shortcomings and suggestions from patients with Type 2 inflammatory diseases.

Interestingly, patients with AD, asthma and CRSwNP shared the same ideas on how to overcome current perceived shortcomings in their care pathways. An overview of the four key shortcomings and suggestions from patients with Type 2 inflammatory diseases is presented in Figure 3. Participants saw EUFOREA as an appropriate organization to implement these suggestions into daily practice. When asked for suggestions, education for both patients, physicians and other healthcare providers was one of the most recurring themes. Patients stated increased knowledge among themselves and caregivers on the following topics as a major opportunity for advancing care: new treatment options and possible side-effects, diagnosis and referral criteria, correct use of medication, prevention of exacerbations, lifestyle advice and control of symptoms. Participants proposed specific training for GPs to ensure faster diagnosis and referral. Many patients were unaware of the existence of online tools, websites and applications for Type 2 inflammation. Others suggested that these tools should be expanded to all Type 2 inflammatory diseases and that their awareness should be raised.

“*Many doctors do not keep up with modern times. I have often been confronted with the “old view” of doctors. Health care is not innovative enough.” (asthma patient)*

“*Education seems like the best route. People can't treat what they don't understand.” (CRSwNP patient)*

“*E-health tools ask about respiratory symptoms (runny nose, how did you sleep…) but nowhere is the question about skin rash asked. It is important for me to be able to report this in the app.” (asthma patient)*

Patients asked for clear guidelines and consistent treatment strategies among different specialists and even among different countries. They recommended the establishment of a list of certified specialised centers in the field of Type 2 inflammation independent of disease location. Participants requested politicians to reach a unified and straightforward agreement on the reimbursement and availability of care for patients with Type 2 inflammatory diseases in Europe. Furthermore, they underlined the need for transparency about the cause of medical costs, that a healthcare system should not make a financial profit and that everyone should enjoy the same insurance model. According to patients, increased awareness could be achieved by addressing specific campaigns on the burden of Type 2 diseases to healthcare providers and the general public. It was repeatedly mentioned that, in contrast to other diseases like cancer, society is not aware of the severity of AD, asthma and CRSwNP. Increased awareness can encourage health policy makers to allocate more resources for the reimbursement and availability of new treatment options, basic skincare, psychological services, training of (para)medics and timely specialist care in order to optimize care and reduce financial costs for the individual patient.

“*Prescribe Dupixent on the NHS, take people out of their everyday misery. Let people breathe and taste again, they will be happier for it!” (CRSwNP patient)*

“*Give doctors the possibility to take more time to listen to their patients.” (AD patient)*

“*Please help to support the dissemination of medication in Denmark there is used in other countries.” (asthma patient)*

Participants pointed out that the need for improvement in multidisciplinary communication and the implementation of a multidisciplinary approach can help to overcome the gaps in holistic care. The necessity for cooperation between psychologists, nurses, lung specialists, skin specialists, ENT specialists and GPs was often highlighted. According to patients, this, together with the questioning of personal preferences and opinions, is a very important step in the pursuit of personalized care.

“*It's nice when all examinations take place in one morning and everything is done in a row with the results of the consultation in the afternoon. It should always be like this.” (CRSwNP patient)*

Finally, EUFOREA and others with similar activities and roles, were seen as important organizations to drive research on the cause of Type 2 inflammation and the connection between lungs, nose and skin. Other suggestions for future research included: new treatment options, the prevention of disease, the genetic transmission of Type 2 inflammation, the effect of COVID-19 on asthma and CRSwNP, the link between food and Type 2 inflammation and a better diagnostic test.

“*EUFOREA could help to improve the process for the patient – such as the process can be coordinate across the different departments and that it all can be like a whole unit.” (asthma patient)*

“*EUFOREA can help to get transparency and conferences on what works and what doesn't. Help to strengthen the cooperation between the different departments. They should look at other health systems in other countries. In Denmark we have become very specialised, e.g. pulmonary medicine - that's all I know and that's ear, nose and throat. Have openness - it goes over each other when they are one body.” (AD patient)*

“*Please do more research. I experience no change in the treatment of my eczema over the last 50 years.” (AD patient)*

“*Find the cause of what's wrong. Treat the cause instead of treating the symptoms.” (asthma patient)*

## Discussion

This preliminary initiative aims to present initial patients' perceptions on the burden of Type 2 diseases, their views on the current shortcomings of care and their perspectives on the improvement of disease management. In-depth and semi-structured interviews of 70 European patients revealed suggestions to overcome current barriers in care pathways to achieve good disease control and better outcomes in the future.

Patients with Type 2 inflammation exhibit a heterogeneous group of symptoms whose impact is underestimated by physicians, society and health policy makers. The classic symptoms of asthma, CRSwNP and AD are well known and widely described in literature. Asthma traditionally presents with shortness of breath, cough, wheeze, limitation during sport and asthma exacerbations (5). Typical CRSwNP symptoms include anosmia, nasal congestion, mucopurulent drainage and facial pressure/headache (3), whereas AD is characterized by itching, redness and inflammation of the skin (13). These factors were identified to contribute to shared overarching and less established consequences such as social isolation, sleep disturbance/fatigue, sexual inhibition, psychosocial distress, functional disturbance and limited activities of daily living (3, 5, 13). Lack of energy and concentration resulted in low productivity and missed workdays. Furthermore, the constant need for adaptations to avoid triggers that could aggravate symptoms led to limited social engagement and reduced sport activities.

Patients who participated in the interviews acknowledged COVID-19 as a source of stress. This was in concordance with a recently published study (27). Firstly, cough, shortness of breath, nasal discharge and headache may be mistaken for symptoms of COVID-19. Secondly, some patients experienced a paralyzing fear of getting infected with the SARS-COVID virus. Thirdly, wearing a face mask caused discomfort and difficulty in breathing. These stressors were linked to asthma and CRSwNP, but not to AD.

Participants agreed on the lack of coordination amongst health care contacts, supporting the results of our previous Unmet Needs in CRSwNP initiative by the EUFOREA Patient Advisory Board (17). Disagreements between physicians, health care inequalities, lack of follow-up, overlooking of comorbidities with focus on one organ, and long waiting lists on the clinics covering the co-morbidities, raised questions about the efficiency and organization of current health care services in different European countries. These patient-perceived barriers do not exist in isolation but interact and influence each other (16). The psychosocial impact of asthma, AD and CRSwNP was often overlooked. While published studies describe the association of depression and anxiety with AD, asthma and CRSwNP, few initiatives regarding mental well-being are being undertaken (3, 5, 13). Some patients were asking for more involvement in treatment decisions, availability of reliable information about their disease and investigation of personal triggers. This patient centered approach can be addressed through the implementation of precision medicine. Patient participation, prediction of treatment success, prevention of disease and personalized care will help to choose the best specific treatment for the global airway and skin of the patient and will help increase patient satisfaction, therapy compliance and disease control (28). Furthermore, a multidisciplinary approach by the implementation of integrated care pathways (ICPs) could help to focus on the prevention, detection, non-pharmacological management and treatment of Type 2 comorbidities (2, 12, 29–32). According to our findings, this holistic approach requires assessment of patients across the following key domains: GPs, psychological services, pulmonologists, dermatologists and ENT specialists. Several multidisciplinary care initiatives for asthma and CRSwNP are being undertaken (30). ARIA (Allergic Rhinitis and its Impact on Asthma) has recently developed integrated care pathways for rhinitis and asthma multimorbidity (31). Nevertheless, ICPs integrating recommendations for the management of asthma, CRSwNP and AD are lacking. Optimizing communication exchange between first-, second- and third-line health care providers will contribute to the continuity of care, multidisciplinary evaluation and regular follow-up of patients. However, further clinical trials to assess multidisciplinary approaches in comparison to standard care are still needed to better document the improvement of overall care and outcome of patients with Type 2 inflammation (2, 30). Additional themes cited by patients for future research projects are mainly the following: connection between lungs-nose-skin, causes of Type 2 inflammation, effect of COVID-19 on chronic respiratory diseases, link between food and Type 2 inflammation and new treatment options.

Despite the broad range of available therapies, patients with asthma, AD and CRSwNP remained uncontrolled with the presence of moderate to severe symptoms that limited their lifestyle. The lack of availability of an effective and preventive therapy treating the root cause of these conditions was one of the most frequently reported frustrations. In contrast to other participants, patients on biologicals reported highly satisfied outcomes alluding to the decrease in severity of disease and the limited frequency of adverse events. Biologics are monoclonal antibodies that, in this case, target Type 2 mediators. Asthma, AD and CRSwNP are inflammatory diseases among subgroups that share common pathophysiological mechanisms which explain their frequent co-existence (1, 2). Targeting the key drivers of Type 2 inflammation has a therapeutic effect on both AD, asthma and CRSwNP (12, 33, 34). Furthermore, biologics have been shown to reduce the need for OCS in patients with severe asthma, CRSwNP and AD (12, 16).

The described adverse effects of systemic corticosteroids and sinus surgery as well as the time- consuming character of current cornerstone treatments underpin the need for new therapeutic strategies that facilitate and improve the lives of patients (12). Considering the many documented benefits and safety profile, many patients are frustrated with the current difficulties in access to biologics. A further key unmet need specific for patients with AD was the lack of reimbursement for topical skincare products, which is an even more important limitation for the care of AD patients in areas with limited resources (35). Participants viewed facilitated access to biological therapies and funding of emollients as possible solutions for disappointing outcomes and high therapy costs. A recent study demonstrated the concurrent efficacy of Dupilumab in AD, asthma and chronic sinonasal conditions, however, the question of whether a unique therapy could lead to a concomitant improvement of all the aforementioned diseases still remains to be further investigated (2, 33). Multiple data already exist showing an improvement in asthma after treatment of CRSwNP and vice versa (1, 2, 7, 12), supporting the link between upper and lower airway diseases (5, 36). In parallel, future research should establish whether biologics should be used in case of moderate CRSwNP and moderate asthma and whether use of biologics can prevent the development of other Type 2 comorbidities and similarly prevent the need for surgical interventions (12).

This patient survey underscores the potential role of EUFOREA, an international non-profit organization, as well as other bodies with similar roles can play in aiming to help healthcare providers at implementing optimal care for patients suffering from chronic respiratory diseases (20, 21) to overcome variously experienced barriers. This can help to create a more empathetic and understanding environment for patients. Lack of knowledge among both clinicians and patients could be addressed by organizing educational programs and training, promoting online applications and providing clear and unbiased information *via* reliable websites. Reliable information is needed to empower patients to self-manage their diseases (20, 21, 31). Clear guidelines on the management of asthma, CRSwNP and atopic dermatitis exist but data have shown that few patients are being treated in line with these guidelines (28, 31). EUFOREA aims to bridge this gap between guidelines and daily practice (17). Physicians are required to receive continuous and high-quality education with the aim of having adequate knowledge of the novel treatment options and optimizing timely diagnosis and referral (30).

Bringing the burden and unmet needs of Type 2 inflammation to the agenda of health policy makers could help to achieve more funding and to emphasize the need for the reorganization of health care systems into harmonized and integrated care in Europe with the purpose of reducing specialist waiting times, facilitating access to biologics and guaranteeing that European citizens enjoy the same rights.

This preliminary report on patient perspectives with moderate to severe Type 2 inflammation diseases might be interesting for several stakeholders. A certain limitation of this survey, though may be the participant selection adopted. Participant recruitment based on severity and follow-up in specialized centers may imply that these patients have more uncontrolled disease than other Type 2 inflammatory patients. On the other hand, these patients are more experienced and more capable of reflecting on their disease journey since they have already passed through the various referral pathways and with yearlong burden. The severity of disease was determined by specialists confirming the presence of moderate to severe disease in previously diagnosed patients within tertiary care referral centers. Patients may have not been able to express themselves fully as the interviews were coordinated from the treating hospital. A key strength of this survey was the inclusion of centers in seven different countries to provide a good view on patients' initial opinions across countries in Europe. Although it should be taken into account that these patients had access to different health care systems which can lead to some differences, however the consistency within the cohort demonstrated some alignment despite different healthcare access and provision of services. A good level of thematic data saturation was achieved, despite the limited number of patients included. Although we followed strict methodological guidelines, thematic analysis is inherently interpretative and thus subject to researcher bias.

## Conclusion

Type 2 inflammation is an underestimated global health problem of increasing prevalence. Patients with asthma, CRSwNP and AD have common unmet needs that need to be addressed by the physician community and health policy makers. This White Paper provides unique recommendations made by patients during this preliminary survey, for the implementation of a holistic disease management in the future, leading to better outcomes of care and cost savings for society. Future work will involve working further and closely with the relevant Patient Advisory Board that support the authors to build further on these insights to help improve care pathways for affected patients with comorbid Type 2 disease.

## Data Availability Statement

The raw data supporting the conclusions of this article will be made available by the authors, without undue reservation.

## Ethics Statement

The study was carried out following the requirements of the Local Ethical Committee guidelines of every participating center (University of Copenhagen, University of Leuven, University of Oldenburg, University of Zurich, London Bridge Hospital, University of Amsterdam, and University of Crete). All participants were briefed about the study objectives and oral informed consent was provided prior to initiating the interview.

## Author Contributions

LD and PH have designed the questions and proposed these questions for approval and discussion by UR, PS-G, VB, EP, WF, and CHo. All authors have revised and approved the content and outcomes of the patient interviews.

## Funding

Funding was provided *via* an unrestricted grant to EUFOREA by Sanofi Genzyme and Regeneron.

## Conflict of Interest

The authors declare that the research was conducted in the absence of any commercial or financial relationships that could be construed as a potential conflict of interest.

## Publisher's Note

All claims expressed in this article are solely those of the authors and do not necessarily represent those of their affiliated organizations, or those of the publisher, the editors and the reviewers. Any product that may be evaluated in this article, or claim that may be made by its manufacturer, is not guaranteed or endorsed by the publisher.

## Abbreviations

ACQ: Asthma Control Questionnaire
AD: Atopic dermatitis
CRSwNP: Chronic Rhinosinusitis with Nasal Polyps
ENT, Ear, Nose: Throat
EUFOREA: European Forum for Research and Education in Allergy and Airways diseases
GP: General Practitioner
ICPs: Integrated care pathways
IGA: Investigator Global Assessment
ILC2: Type 2 Innate Lymphoid Cells
OCS: Oral Corticosteroids
PAB: Patient Advisory Board
SCORAD: SCORing Atopic Dermatitis
SNOT22: Sino-Nasal Outcome Test-22
Th2: T helper 2.

## References

[1] McCormick JP Lee JT. Insights into the implications of coexisting type 2 inflammatory diseases. J Inflamm Res. (2021) 14:4259–66. 10.2147/JIR.S311640 34511966 PMC8416183

[2] Hassoun D Malard O Barbarot S Magnan A Colas L. Type 2 immunity-driven diseases: towards a multidisciplinary approach. Clin Exp Allergy. (2021) 51:1538–52. 10.1111/cea.14029 34617355 PMC9292742

[3] Fokkens WJ Lund VJ Hopkins C Hellings PW Kern R Reitsma S. European position paper on rhinosinusitis and nasal polyps 2020. Rhinology. (2020) 58:1–464. 10.4193/Rhin20.401 32077450

[4] Silverberg JI Hanifin JM. Adult eczema prevalence and associations with asthma and other health and demographic factors: a US population-based study. J Allergy Clin Immunol. (2013) 132:1132–8. 10.1016/j.jaci.2013.08.031 24094544

[5] Global Initiative for Asthma,. Global Strategy for Asthma Management Prevention. (2021). Available online at: www.ginasthma.org (accessed December 29, 2021).

[6] Khan A Vandeplas G Huynh TMT Joish VN Mannent L Tomassen P. The Global Allergy and Asthma European Network (GALEN) rhinosinusitis cohort: a large European cross-sectional study of chronic rhinosinusitis patients with and without nasal polyps. Rhinology. (2019) 57:32–42. 10.4193/Rhin17.255 29911211

[7] Bachert C Han JK Desrosiers M Hellings PW Amin N Lee SE. Efficacy and safety of dupilumab in patients with severe chronic rhinosinusitis with nasal polyps (LIBERTY NP SINUS-24 and LIBERTY NP SINUS-52): results from two multicentre, randomised, double-blind, placebo- controlled, parallel-group phase 3 trials. Lancet. (2019) 394:1638–50. 10.1016/S0140-6736(19)31881-1 31543428

[8] Silverberg JI Gelfand JM Margolis DJ Boguniewicz M Fonacier L Grayson MH. Association of atopic dermatitis with allergic, autoimmune, and cardiovascular comorbidities in US adults. Ann Allergy Asthma Immunol. (2018) 121:604–12. 10.1016/j.anai.2018.07.042 30092266 PMC13217624

[9] Tan BK Chandra RK Pollak J Kato A Conley DB Peters AT. Incidence and associated premorbid diagnoses of patients with chronic rhinosinusitis. J Allergy Clin Immunol. (2013) 131:1350–60. 10.1016/j.jaci.2013.02.002 23541327 PMC3788631

[10] Van der Veen J Seys SF Timmermans M Levie P Jorissen M Fokkens WJ. Real-life study showing uncontrolled rhinosinusitis after sinus surgery in a tertiary referral centre. Allergy. (2017) 72:282–90. 10.1111/all.12983 27392210 PMC5248621

[11] Van Bulck P Cools L Soumya MS Nyembue DT Kabobo P Zhang L. A multicenter real-life study on the multiple reasons for uncontrolled allergic rhinitis. Int Forum Allergy Rhinol. (2021) 11:1452–60. 10.1002/alr.22808 34259380

[12] Fokkens WJ Lund V Bachert C Mullol J Bjermer L Bousquet J. EUFOREA consensus on biologics for CRSwNP with or without asthma. Allergy. (2019) 74:2312–9. 10.1111/all.13875 31090937 PMC6972984

[13] Silverberg JI. Comorbidities and the impact of atopic dermatitis. Ann Allergy Asthma Immunol. (2019) 123:144–51. 10.1016/j.anai.2019.04.020 31034875

[14] Lourijsen ES Fokkens WJ Reitsma S. Direct and indirect costs of adult patients with chronic rhinosinusitis with nasal polyps. Rhinology. (2020) 58:213–7. 10.4193/Rhin19.468 32415826

[15] Dierick BJH van der Molen T Flokstra-de Blok BMJ Muraro A Postma MJ Kocks JWH. Burden and socioeconomics of asthma, allergic rhinitis, atopic dermatitis and food allergy. Expert Rev Pharmacoeconomics Outcomes Res. (2020) 20:437–53. 10.1080/14737167.2020.1819793 32902346

[16] Majellano EC Clark VL Winter NA Gibson PG McDonald VM. Approaches to the assessment of severe asthma: Barriers and strategies. J Asthma Allergy. (2019) 12:235–51. 10.2147/JAA.S178927 31692528 PMC6712210

[17] Claeys N Teeling MT Legrand P Poppe M Verschueren P De Prins L. Patients unmet needs in chronic rhinosinusitis with nasal polyps care: a patient advisory board statement of EUFOREA. Front Allergy. (2021) 2:761388. 10.3389/falgy.2021.761388 35386961 PMC8974789

[18] Vennik J Eyles C Thomas M Hopkins C Little P Blackshaw H. Chronic rhinosinusitis: a qualitative study of patient views and experiences of current management in primary and secondary care. BMJ Open. (2019) 9. 10.1136/bmjopen-2018-022644 31015263 PMC6501991

[19] Zuberbier T Orlow SJ Paller AS Taïeb A Allen R Hernanz-Hermosa JM. Patient perspectives on the management of atopic dermatitis. J Allergy Clin Immunol. (2006) 118:226–32. 10.1016/j.jaci.2006.02.031 16815160

[20] Pugin B Deneyer L Bachert C Alobid I Bousquet J de Carlo G. Patient advisory board for chronic rhinosinusitis–a euforea initiative. Rhinology. (2019) 57:331–5. 10.4193/Rhin19.012 30963145

[21] Hellings PW Borrelli D Pietikainen S Agache I Akdis C Bachert C. European summit on the prevention and self-management of chronic respiratory diseases: report of the European Union Parliament Summit (29 March 2017). Clin Transl Allergy. (2017) 7. 10.1186/s13601-017-0186-3. [Epub ahead of print]. 29299230 PMC5745781

[22] O'Brien BC Harris IB Beckman TJ Reed DA Cook DA. Standards for reporting qualitative research: a synthesis of recommendations. Acad Med. (2014) 89:1245–51. 10.1097/ACM.0000000000000388 24979285

[23] Wollenberg A Barbarot S Bieber T Christen-Zaech S Deleuran M Fink-Wagner A. Consensus-based European guidelines for treatment of atopic eczema (atopic dermatitis) in adults and children: part I. J Eur Acad Dermatol Venereol. (2018) 32:657–82. 10.1111/jdv.14891 29676534

[24] Kallio H Pietilä A-M Johnson M Kangasniemi M. Systematic methodological review: developing a framework for a qualitative semi-structured interview guide. J Adv Nurs. (2016) 72:2954–65. 10.1111/jan.13031 27221824

[25] Braun V Clarke V. Using thematic analysis in psychology. Qual Res Psychol. (2006) 3:77–101. 10.1191/1478088706qp063oa

[26] Dierckx de Casterle B Gastmans C Bryon E Denier Y. QUAGOL: a guide for qualitative data analysis. Int J Nurs Stud. (2012) 49:360–71. 10.1016/j.ijnurstu.2011.09.012 21996649

[27] Wei L Islam JY Mascareno EA Rivera A Vidot DC Camacho-Rivera M. Physical and mental health impacts of the COVID-19 pandemic among US adults with chronic respiratory conditions. J Clin Med. (2021) 10:3981. 10.3390/jcm10173981 34501426 PMC8432199

[28] Hellings PW Fokkens WJ Bachert C Akdis CA Bieber T Agache I. Positioning the principles of precision medicine in care pathways for allergic rhinitis and chronic rhinosinusitis–A EUFOREA-ARIA-EPOS-AIRWAYS ICP statement. Allergy. (2017) 72:1297–305. 10.1111/all.13162 28306159

[29] Campbell H Hotchkiss R Bradshaw N Porteous M. Integrated care pathways. Br Med J. (1998) 316:133–7. 10.1136/bmj.316.7125.133 9462322 PMC2665398

[30] Vlastos I Mullol J Hox V Doulaptsi M Seys S Hellings P. Multidisciplinary Care for Severe or Uncontrolled Chronic Upper Airway Diseases n.d. 33791881 10.1007/s11882-021-01004-z

[31] Bousquet JJ Schünemann HJ Togias A Erhola M Hellings PW Zuberbier T. Next-generation ARIA care pathways for rhinitis and asthma: a model for multimorbid chronic diseases. Clin Transl Allergy. (2019) 9. 10.1186/s13601-019-0289-0 31516692 PMC6734297

[32] Backer V Aanaes K Hansen S Petersen J von Buchwald C. Global airways–a novel standard test for asthma, allergic rhinitis, and chronic rhinosinusitis (STARR-15). Rhinology. (2022) 60:63–72. 10.4193/Rhin21.195 35174371

[33] Boguniewicz M Beck LA Sher L Guttman-Yassky E Thaçi D Blauvelt A. Dupilumab improves asthma and sinonasal outcomes in adults with moderate to severe atopic dermatitis. J Allergy Clin Immunol Pract. (2021) 9:1212–23. 10.1016/j.jaip.2020.12.059 33453450

[34] Gandhi NA Bennett BL Graham NMH Pirozzi G Stahl N Yancopoulos GD. Targeting key proximal drivers of type 2 inflammation in disease. Nat Rev Drug Discov. (2016) 15:35–50. 10.1038/nrd4624 26471366

[35] Schmid-Grendelmeier P Takaoka R Ahogo KC Belachew WA Brown SJ Correia JC. Position statement on atopic dermatitis in Sub-Saharan Africa: current status and roadmap. J Eur Acad Dermatol Venereol. (2019) 33:2019–28. 10.1111/jdv.15972 31713914 PMC6899619

[36] Hens G Vanaudenaerde BM Bullens DMA Piessens M Decramer M Dupont LJ. Sinonasal pathology in nonallergic asthma and COPD: “United airway disease” beyond the scope of allergy. Allergy. (2008) 63:261–7. 10.1111/j.1398-9995.2007.01545.x 18053011

