# Corrigendum on: White paper on European patient needs and suggestions on chronic type 2 inflammation of airways and skin by EUFOREA

**DOI:** 10.3389/falgy.2022.1001078

**Published:** 2022-09-09

**Authors:** Louise De Prins, Ulrike Raap, Tara Mueller, Peter Schmid-Grendelmeier, Christiane H. Haase, Vibeke Backer, Wytske Fokkens, Linda B. Benoist, Emmanuel Prokopakis, Maria Doulaptsi, Claire Hopkins, Nele Claeys, Thijs Teeling, Lindsay Cypers, Leen Cools, Leif H. Bjermer, Zuzana Diamant, Ulrich Wahn, Glenis Scadding, Claus Bachert, Peter Walther, Sunni R. Patel, Elizabeth Van Staeyen, Peter Hellings

**Affiliations:** ^1^ Department of Otorhinolaryngology, EUFOREA Scientific Expert Team Members, Brussels, Belgium; ^2^ Department of Otorhinolaryngology, Head and Neck Surgery, Universitair Ziekenhuis Leuven, Leuven, Belgium; ^3^ Division of Experimental Allergology and Immunodermatology, University of Oldenburg, Oldenburg, Germany; ^4^ Department of Dermatology, Klinikum Oldenburg, University of Oldenburg, Oldenburg, Germany; ^5^ Department of Dermatology, Allergy Unit, University Hospital of Zurich, Zurich, Switzerland; ^6^ Department of Otorhinolaryngology Head and Neck Surgery and Audiology, Rigshospitalet, Copenhagen University, Copenhagen, Denmark; ^7^ Department of Otorhinolaryngology, Head / Neck Surgery, Academic Medical Center (AMC), Amsterdam, Netherlands; ^8^ Department of Otorhinolaryngology-Head and Neck Surgery, University of Crete School of Medicine, Heraklion, Crete, Greece; ^9^ Department of Otorhinolaryngology, Guy’s and St Thomas’ Hospital, London Bridge Hospital, London, United Kingdom; ^10^ EUFOREA Patient Advisory Board Chairs; ^11^ Upper Airways Research Laboratory, Department of Otorhinolaryngology, University of Ghent, Ghent, Belgium

**Keywords:** atopic dermatitis (AD), asthma, chronic rhinosinusitis, nasal polyps, type 2 inflammation, quality of life, unmet needs

A corrigendum on White paper on European patient needs and suggestions on chronic type 2 inflammation of airways and skin by EUFOREA By De Prins L, Raap U, Mueller T, Schmid-Grendelmeier P, Haase CH, Backer V, Fokkens W, Benoist LB, Prokopakis E, Hopkins C, Claeys N, Teeling T, Cools LCL, Bjermer LH, Diamant Z, Wahn U, Scadding G, Bachert C, Patel SR, Van Staeyen E and Hellings P. (2022). Front. Allergy. 3:889221. doi: 10.3389/falgy.2022.889221

**Error in author list**

In the published article, there was an error in the author list, and authors Maria Doulaptsi and Peter Walther were erroneously excluded. The corrected author list appears below.

Louise De Prins^1,2^

Ulrike Raap^3,4^

Tara Mueller^3,4^

Peter Schmid-Grendelmeier^1,5^

Christiane H. Haase^6^

Vibeke Backer^1,6^

Wytske Fokkens^1,7^

Linda B. Benoist^7^

Emmanuel Prokopakis^1,8^

Maria Doulaptsi^8^

Claire Hopkins^1,9^

Nele Claeys^10^

Thijs Teeling^10^

Lindsay Cypers^1^*

Leen Cools^1,2^

Leif H. Bjermer^1^

Zuzana Diamant^1^

Ulrich Wahn^1^

Glenis Scadding^1^

Claus Bachert^11^

Peter Walther^1^

Sunni R. Patel^1^

Elizabeth Van Staeyen^1^

Peter Hellings^1,^^2,^^7,^^11^

The authors apologize for this error and state that this does not change the scientific conclusions of the article in any way. The original article has been updated.

