# Supplementary figures and images for: White Paper on European Patient Needs and Suggestions on Chronic Type 2 Inflammation of Airways and Skin by EUFOREA
Source: Front Allergy. 2022 Jun 2;3:889221. doi: 10.3389/falgy.2022.889221 (PMC9234878; doi:10.3389/falgy.2022.889221)

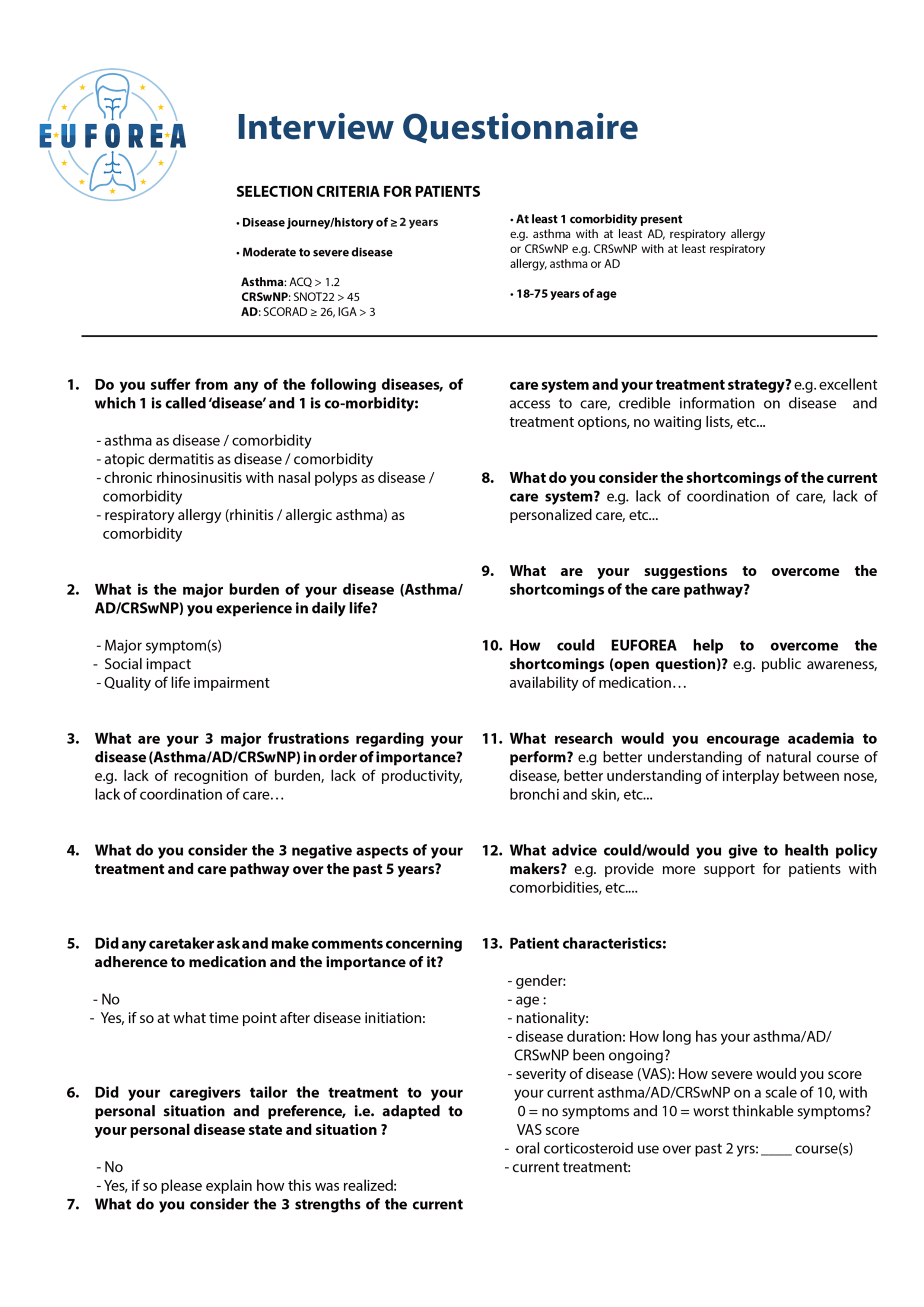

Supplement: Supplementary file 1 [file Figure_1.tif]
